# Sleep duration and incidence of lung cancer in ageing men

**DOI:** 10.1186/1471-2458-14-295

**Published:** 2014-03-31

**Authors:** Maria K Luojus, Soili M Lehto, Tommi Tolmunen, Arja T Erkkilä, Jussi Kauhanen

**Affiliations:** 1 Institute of Public Health and Clinical Nutrition, University of Eastern Finland, P.O. Box 162770211 Kuopio, Finland; 2 Department of Psychiatry, Kuopio University Hospital, P.O. Box 10070029 KYS Kuopio, Finland; 3 University of Eastern Finland, P.O. Box 162770211 Kuopio, Finland

**Keywords:** Prospective cohort study, Sleep duration, Lung cancer

## Abstract

**Background:**

Previous studies have suggested an association between sleep duration and cancer. However, the information on sleep duration regard to risk of lung cancer is scanty.

**Methods:**

Analysed data comprised prospective population-based cohort of 2586 men (aged 42–60 years) from Eastern Finland. Baseline survey and clinical examinations took place 1984–1989, and diagnosed lung cancers were obtained until the end of 2011 through linkage with the Finnish Cancer Registry. Self-reported sleep was categorized as ≤6.5 h, 7–7.5 h, and ≥8 h. Subjects with prior history of cancer or psychotropic medication (hypnotics or sedatives) were excluded from the analyses. Cox proportional hazards models with adjustments for possible confounders were used to examine the association.

**Results:**

Significant association between sleep duration and increased lung cancer risk was observed after adjustments for age, examination years, cumulative smoking history, family cancer history and Human Population Laboratory Depression scale scores (HR 2.12, 95% CI 1.17-3.85 for ≤6.5 h sleep, and HR 1.88, 95% CI 1.09-3.22 for ≥8 h sleep). Associations were even stronger among current smokers (HR 2.23, 95% CI 1.14-4.34 for ≤6.5 h sleep, and HR 2.09, 95% CI 1.14-3.81 for ≥8 h sleep). After further adjustments for alcohol consumption, physical activity, body mass index, marital status, education years, night work, employment status, asthma and chronic bronchitis, the association remained significant both in the whole study population and among smokers. When cumulative smoking history was replaced by current smoking in the adjustments, the increased risk was limited to those who slept <6.5 h.

**Conclusions:**

Sleep duration of less than 7–7.5 hours or more than 7–7.5 hours associates with increased lung cancer risk. The physiological factors underlying the association are complex, and they may relate to melatonin excretion patterns, low-grade inflammation in cancer development process or disruptions in circadian rhythmicity.

## Background

Lung cancer is estimated to account for 8.3% of men’s cancer cases globally [1]. Among all types of cancers worldwide, lung cancer incidence is highest [2] and in Finland it comes second [3] after prostate cancer. Previous studies [4,5] have identified numerous factors to increase risk of lung cancer, such as smoking history, previous lung disease (bronchitis, asthma, pneumonia, chronic obstructive pulmonary disease, cystic fibrosis, or obstructive sleep apnoea), environmental exposures, and genetic predisposition.

In addition to established risk factors, it has been suggested that sleep duration may associate with cancer development. Both short and long sleep duration are proposed to be a determinants of increased cancer risk [6]. One possible factor underlying the association is melatonin hormone, which represents the most stable and reliable biomarker of the central circadian pacemaker [7,8], and may play a role in lung cancer tumor growth [9,10]. This suggestion is supported by the observations of severe disruptions in circadian rhythms among lung cancer patients [11-15]. These circadian rhythm disruptions consists of a loss of rhythmicity in neuroendocrine and immune parameters [11], as well as disturbed daily sleep-activity cycles [12-15]. Our focus is on altered sleep duration, which can be one of the symptoms of a circadian rhythm sleep disorders [7].

Most of previous studies have proposed nightly sleep less than 6–7 hours to increase risk of cancer in general [16], and specifically prostate cancer [17], breast cancer [18-20] and colorectal adenoma [21]. One recently conducted prospective follow-up study found U-shaped association between sleep duration and colorectal cancer incidence in postmenopausal women [22]. Furthermore, circadian rhythm sleep disorders [8,23] have shown the relation between sleep and increased cancer risk.

Our focus is on the relationship between lung cancer and altered sleep duration. To the best of our knowledge, there are no previous prospective cohort studies on this topic. To assess the possible association between sleeping hours and increased lung cancer risk, we conducted the prospective cohort study among 2586 ageing men from Eastern Finland.

## Methods

### Study population

The prospective cohort Kuopio Ischemic Heart Disease Study (KIHD) participants were a randomly selected sample from general population in Eastern Finland [24]. Baseline examinations during 1984–1989 were conducted in 2682 men (82.9% of those invited) aged 42–60 years, living in Kuopio or surrounding rural area. Men having cancer history (n = 51, 1.9%), or using hypnotics or sedatives (n = 45, 1.7%) at baseline were excluded, leaving total of 2586 respondents. Participants provided written informed consent after full explanation of study, and the Research Ethics Committee of Kuopio University approved the study protocol.

### Outcome

Diagnosed lung cancers (n = 81, 3.1%) occurring from two years after baseline until end of 2011 were included. To rule out the reverse-causation we restricted our sample to those who had had cancer at least two years at the baseline. Median follow-up time was 23 years (25^th^-75^th^ percentiles were 18–25 years). Lung cancer diagnoses were ascertained by the individual social security number linkage with the Finnish Cancer Registry. Diagnoses were classified according to International Classification of Diseases (ICD-8, −9, and −10).

### Baseline measures

#### Sleep

Self-administered questionnaires were recorded by participants and checked by an interviewer. Sleep duration was asked: ‘How many hours do you usually sleep at night?’ Response alternatives were: <6 h, 6.5, 7, 7.5, 8, 8.5, 9, 9.5, and >10 h. Crude lung cancer incidence rate ratios were lowest in 7 h (1.21 cases/1 000 person years) and 7.5 h (0.90 cases/1 000 person years) of sleep, and thus sleep duration was divided into three categories: ≤6.5 h, 7–7.5 h, and ≥8 h. Comparable reference sleep duration category is presented in previous cancer and sleep studies [25]. Other sleep questions were: ‘Do you have nightly breathing disruptions?’ (yes/no), and ‘How often do you have difficulties to fall asleep, or staying asleep?’ (never or seldom/sometimes/frequently).

#### Depressive symptoms

Depressive symptoms were assessed with the 18-item Human Population Laboratory Depression Scale HPL; [26]. Scale items included mood disturbance, negative self-concept, energy loss, poor appetite, concentration difficulty, and psychomotor agitation. The scale was developed especially for screening general population samples, and it also conceptually resembles other brief symptom checklists such as the Centre for Epidemiological Studies Depression Scale CES-D; [27,28]. The HPL Depression score is generated by assigning one point for each true or false answer that is indicative for depression (range 0–18). To avoid collinearity, insomnia was excluded from the scale.

#### Health and sociodemographic background

Participants completed questionnaires concerning medication (hypnotics, sedatives and antidepressants), and history of physician-diagnosed illnesses (cancers, chronic bronchitis, asthma, and family cancer history). To assess physical activity, the 12-Month Physical Activity questionnaire [29] was applied. The checklist included the most common physical activities (walking, jogging, skiing, bicycling, swimming, games) of Finnish middle-aged men. For each activity performed, the subjects were asked to record the frequency, average duration and intensity. The energy expenditure from physical activity was expressed as kcal/day.

Body mass index (BMI) was computed as the ratio of weight (kilograms) to the square of height (meters). Current smoking behavior was assessed with three questions: 1) Have you ever smoked? Yes/no, 2) Do you smoke daily during last year? Yes/no, and 3) When have you smoked last time? (Less than month is defined as current smoker). Cumulative smoking history (pack-years) was estimated as product of years smoked and number of tobacco products smoked daily at the time of examination. Alcohol consumption (g/wk) was assessed with a structured quantity-frequency method using the Nordic Alcohol Consumption Inventory for drinking behavior over previous 12 months [30]. Respondents were asked for total years of education, marital status (married or living with spouse vs. living alone), working time (day shift vs. night shift), and employment status (employed vs. unemployed or retired).

### Statistics

According to variable type and distribution, baseline variables were displayed by the sleep categories with Kruskall-Wallis or χ^2^ test p-values. Moreover, correlation matrix for the continuous variables was made. Kaplan-Meier survival curves (Mantel-Cox log-rank test) differed significantly (p-value 0.04) between sleep duration categories allowing Cox proportional hazards model application. Covariates were selected based on factors affecting sleep [31] and lung cancer development [4,5]. To compute hazard ratios (HR) and confidence intervals (95% CI), we first built a Model^a^ that was adjusted for age and examination years. Model^a^ was further adjusted for cumulative smoking history (pack-years) (Model^b^), and family cancer history and HPL scale scores (Model^c^). Model^c^ was further adjusted for alcohol consumption, physical activity and BMI (Model^d^), education years, marital status, working time and employment status (Model^e^), and asthma and chronic bronchitis (Model^f^). Stratified analyses for whole study population and smokers were formed for following reasons: 1) Smoking is an established risk for lung cancer [32], and 2) We observed substantially more new lung cancer cases among smokers within ≤6.5 h, 7–7.5 h and ≥8 h sleep groups (cancer cases 18, 18, and 32, respectively) than among nonsmokers (cancer cases 3, 6, and 4, respectively). Analyses were conducted with SPSS software (IBM Company, SPSS Statistics version 19.0, United States).

## Results

Table 1 displays baseline characteristics according to sleep categories. Smoking, BMI, education years, HPL scale scores, night work, employment status, chronic bronchitis, frequent insomnia and nightly breathing disruptions associated with sleep duration. Table 2 demonstrates the correlations between continuous baseline variables. Education years and HPL scores had significant correlations with all other continuous variables including sleeping hours. Furthermore, sleeping hours correlated with cumulative smoking history.

**Table 1:** Baseline characteristics according to sleep duration in ageing men

	**Sleeping hours**			**p-value**
	**≤6.5 h**	**7-7.5 h**	**≥8 h**	
	**(n = 501)**	**(n = 1066)**	**(n = 1019)**	
Age (years)	54.3 (48.9-54.5)	54.3 (48.8-54.5)	54.3 (54.2-54.6)	0.075^a^
Smoking prevalence (%)	194 (38.7)	315 (29.5)	322 (31.6)	<0.001^b^
Smoking (pack-years)	0 (0–378.0)	0 (0–81.9)	0 (0–204.0)	0.001^a^
Alcohol (g/wk)	34.2 (6.6-104.4)	31.9 (6.6-86.3)	27.8 (4.7-16.2)	0.208^a^
Physical activity (kcal/d)	80.1 (27.6-201.4)	84.9 (30.8-199.2)	85.0 (27.2-344.0)	0.831^a^
Body mass index (kg/m^2^)	26.6 (24.5-29.5)	26.2 (24.3-28.3)	26.8 (24.7-29.4)	<0.001^a^
Education (years)	8.0 (6.0-10)	8.0 (6.0-10.0)	8.0 (6.0-9.0)	<0.001^a^
HPL scores ≥5 (%)	68 (14.0)	84 (8.0)	79 (7.9)	<0.001^b^
Family cancer history (%)	119 (23.8)	259 (23.8)	248 (24.3)	0.965^b^
Marital status (alone %)	35 (7.0)	62 (5.8)	73 (7.2)	0.425^b^
Night work (%)	113 (22.6)	160 (15.0)	181 (17.8)	0.001^b^
Unemployed/retired (%)	147 (29.3)	255 (23.9)	379 (37.2)	<0.001^b^
Asthma (%)	15 (3.0)	34 (3.2)	43 (4.2)	0.341^b^
Chronic bronchitis (%)	48 (9.6)	65 (6.1)	69 (6.8)	0.040^b^
Frequent insomnia (%)	161 (33.0)	115 (10.9)	91 (9.1)	<0.001^b^
Breathing disruptions (%)	84 (17.0)	116 (11.0)	119 (11.8)	0.003^b^

Values are given as median (25^th^-75^th^ percentile), or as n and proportion (%).

^a^Kruskal-Wallis median test.

^b^χ ^2^ test.

Abbreviations: *HPL* Human Population Laboratory Depression Scale.

**Table 2:** Correlations between the continuous baseline variables

	**Smoking**	**Alcohol**	**Physical activity**	**Body mass index**	**Education**	**HPL scores**	**Sleeping hours**
	**(pack-years)**	**(g/week)**	**(kcal/d)**	**(kg/m**^**2**^**)**	**(years)**		
Age (years)	0.002 (0.931)	−0.106 (<0.001)	0.001 (0.990)	0.030 (0.129)	−0.297 (<0.001)	0.074 (<0.001)	0.029 (0.138)
Smoking (pack-years)	-	0.258 (<0.001)	−0.184 (<0.001)	−0.145 (<0.001)	−0.080 (<0.001)	0.090 (<0.001)	−0.043 (0.030)
Alcohol (g/week)	-	-	0.016 (0.418)	0.083 (<0.001)	0.116 (<0.001)	0.046 (0.021)	−0.027 (0.177)
Physical activity (kcal/d)	-	-	-	0.014 (0.467)	0.154 (<0.001)	−0.085 (<0.001)	−0.006 (0.750)
Body mass index (kg/m^2^)	-	-	-	-	−0.056 (0.005)	0.051 (0.011)	0.039 (0.051)
Education (years)	-	-	-	-	-	−0.098 (<0.001)	−0.064 (0.001)
HPL scores	-	-	-	-	-	-	−0.056 (0.005)

Values are given as Spearman’s correlation coefficient (p-values).

Abbreviations: *HPL* Human Population Laboratory Depression Scale.

We carried out proportional hazards analysis with adjustments for possible confounders. As a result, approximately twofold risk for lung cancer was observed in the ≤6.5 h and ≥8 h sleep groups both in the whole study population and among smokers (Table 3). To assess the effect of current smoking, we performed Cox proportional hazards analysis adjusted for age, examination year and current smoking in the whole study population. Association between ≥8 h sleep and lung cancer lost significance (HR 1.54, 95% CI 0.92-2.58), but remained in the ≤6.5 h sleep (HR 1.82, 95% CI 1.01-3.28).

**Table 3:** Men’s lung cancer Hazard Ratios (95% Confidence Interval) according to sleep duration and smoking status

**Whole study population**	**Sleeping hours**				
	**≤6.5 h**		**7-7.5 h**	**≥8 h**	
	**n = 501 (21 cancer)**		**n = 1066 (24 cancer)**	**n = 1019 (36 cancer)**	
	**HR**	**95% CI**	**HR**	**HR**	**95% CI**
Model^a^	2.06	1.14-3.69	1.0	1.68	1.01-2.83
Model^b^	1.95	1.08-3.51	1.0	1.79	1.06-3.02
Model^c^	2.12	1.17-3.85	1.0	1.88	1.09-3.22
Model^d^	2.01	1.09-3.71	1.0	1.96	1.14-3.38
Model^e^	2.14	1.17-3.90	1.0	1.80	1.04-3.09
Model^f^	1.96	1.07-3.58	1.0	1.82	1.06-3.11
**Smokers**	**n = 194 (18 cancer)**		**n = 315 (18 cancer)**	**n = 322 (32 cancer)**	
Model^a^	2.01	1.04-3.87	1.0	1.80	1.01-3.21
Model^b^	2.04	1.06-3.92	1.0	1.95	1.09-3.49
Model^c^	2.23	1.14-4.34	1.0	2.09	1.14-3.81
Model^d^	2.21	1.11-4.04	1.0	2.22	1.21-4.09
Model^e^	2.20	1.12-4.34	1.0	2.02	1.10-3.71
Model^f^	2.15	1.09-4.22	1.0	2.04	1.12-3.74

Model^a^: Adjusted for age and examination years.

Model^b^: Adjusted for age, examination years and cumulative smoking history (pack-years).

Model^c^ : Adjusted for age, examination years, cumulative smoking history (pack-years), family cancer history and Human Population Laboratory depression scale scores.

Model^d^: Model^c^ further adjusted for: Alcohol consumption (g/wk), physical activity (kcal/day) and body mass index (kg/m^2^).

Model^e^: Model^c^ further adjusted for: Marital status, education years, night work, and employment status.

Model^f^: Model^c^ further adjusted for: Asthma and chronic bronchitis.

We further examined other sleep variables. Nightly breathing disruptions and frequent insomnia were not associated with incidence of lung cancer (age-adjusted HR 1.36, 95% CI 0.75-2.47 and HR 1.16, 95% CI 0.72-1.89, respectively). These conditions cumulated in men with sleep of 6.5 h or less (Table 1).

## Discussion

### Summary of main findings

Sleep duration of less than 7–7.5 hours or more than 7–7.5 hours associates with increased lung cancer risk in ageing men irrespective of age, cumulative smoking history, family cancer history, night work, health behavior, sociodemographic characteristics and previous inflammatory lung diseases. However, adjusting for current smoking instead of cumulative smoking history, limited the increased risk to the men with nightly sleep 6.5 h or less.

### Comparison with previous literature

In this prospective population-based study we observed an association between sleep duration and moderately increased lung cancer risk. Comparable association has been found in the risk of colorectal cancer [22] and in all-cause mortality [31]. Recently conducted meta-analysis concerning sleep duration and breast, prostate, endometrial, thyroid, ovarian and colorectal cancer risk [25] suggests that sleep duration and cancer are not connected to each other. Nevertheless, in the subgroup analyses (3 studies included in) the researchers found higher colorectal cancer risk in the long sleep group. However, the number of sleep duration and cancer risk studies is small, as well as the number of studies concerning different cancer types.

Smoking is well-known risk for lung cancer [32], which was clearly demonstrated also in our study as a substantially higher incidence of lung cancer cases among smokers. Smoking is also connected with depression [33] and sleep disorders [34]. Regular smoking impairs the nightly sleep structure due to the biological effects of nicotine [35], which was observed in our study too.

In addition to smoking, diet and body weight affect both sleep and cancer. In the sleep perspective, the ideal body mass index goes hand in hand with sleep of approximately 7 hours [36], whereas cancer can induce cachexia including loss of appetite, weight loss and hypermetabolism [37].

Overall, a number of possible pathways underlying the association between sleep duration and cancer have been proposed. They relate among other things to clock-gene deregulation induced tumor genesis and progression of cancer [38], immunosuppression due to deprivation or restriction of sleep [39], and altered melatonin secretion patterns, such as timing [40,41], amount [42,43], and secretion duration [44]. Melatonin has oncostative properties in tumors, including antioxidant effects, modulation of cell cycle and apoptosis, inhibition of telomerase activity and metastasis, stimulation of cell differentiation, and prevention of chronodisruption CD; [45].

Lung cancer patients have frequently CD [13,14] with severe alterations of neuroendocrine and immunological factors [11]. However, shift work with CD has been classified as a probable, group 2A carcinogen by the International Agency for Research on Cancer [46]. Shift work can lead to CD including physiological, endocrinological, and sleep-wake cycle alterations, which may increase the risk for breast, endometrial, prostate and colon cancer [8,23].

In the sleep perspective, circadian rhythm sleep disorders (CRSD) include a variety of conditions such as: time zone change, shift work sleep disorder, irregular sleep-wake rhythm, free-running disorder, delayed sleep phase disorder, and advanced sleep phase disorder [7,47]. CRSDs relate to both timing and duration of sleep, in other words, ‘The essential feature of CRSDs is a persistent or recurrent pattern of sleep disturbance primarily due to alterations in the circadian timekeeping system or a misalignment between the endogenous circadian rhythm and exogenous factors that affect the timing or duration of sleep [7]’. We were interested in altered sleep duration, which can be one symptom of a circadian rhythm sleep disorders [7].

Inflammatory processes are one etiological factor in the lung cancer development [48]. Sleep and immunity have a complex relation, where poor sleep may suppress immunity [39], and in turn, chronic low-grade inflammation may induce sleepiness, fatigue and reduced quality of sleep [49].

### Strengths and limitations

Our study comprised regionally representative sample of ageing men with high participation rate. The follow-up information on lung cancer diagnoses was inclusive. All cancer diseases diagnosed in Finland since 1953 have been registered to the Finnish Cancer Registry, which coverage is virtually complete without loss to follow-up [50]. We were able to measure various covariates, such as body mass index and depressive symptoms, which affect sleep and/or lung cancer risk. Also the exclusion of those having cancer diagnosis at baseline or within the two years following improved assessment of association, because exposures were measured before disease onset. To avoid confounding effect, exclusion of hypnotics and sedative users were made at the baseline.

Nevertheless, following limitations in the study need to be considered while interpreting the results. (i) Our observations cannot be generalized to women and younger men. (ii) We were not able to measure all known lung cancer risk factors, like environmental exposures, as well as the changes in sleep duration and health behavior during the follow up time. (iii) Information on sleeping hours can lead to misclassification, because self-reported hours tend to be greater than objectively measured hours [51]. Furthermore, sleep was measured as a single time point measurement. (iv) The number of new lung cancer cases was small during follow-up time. (v) We were not able to assess the effect of melatonin intake. However, the use of melatonin was low in Finland during 1997-2007 [52].

## Conclusions

Sleep duration of less than 7–7.5 hours or more than 7–7.5 hours associates with increased lung cancer risk irrespective of age, health behavior, previous inflammatory lung diseases and sociodemographic status. However, adjusting for current smoking instead of cumulative smoking history, limited the increased risk to the men with nightly sleep 6.5 h or less. The physiological factors underlying the association are complex, and may relate to melatonin excretion patterns, low-grade inflammation in cancer development process, or disruptions in circadian rhythmicity.

## Abbreviations

BMI: Body Mass Index; CI: Confidence Interval; CD: Chronodisruption; CRDS: Circadian Rhythm Sleep Disorders; HR: Hazard Ratio; HPL: Human Population Laboratory Depression Scale; ICD: International Classification of Diseases; KIHD: Kuopio Ischemic Heart Disease Study.

## Competing interests

The authors declare that they have no competing interests.

## Authors’ contributions

ML carried out data analyses and drafted the manuscript. SL, TT, AE, and JK participated to data analyses, and drafting and revising the manuscript. All authors read and approved the final manuscript.

## Pre-publication history

The pre-publication history for this paper can be accessed here:

http://www.biomedcentral.com/1471-2458/14/295/prepub
